# Subsurface Super-resolution Imaging of Unstained Polymer Nanostructures

**DOI:** 10.1038/srep28156

**Published:** 2016-06-29

**Authors:** Ben E. Urban, Biqin Dong, The-Quyen Nguyen, Vadim Backman, Cheng Sun, Hao F. Zhang

**Affiliations:** 1 Northwestern University, Department of Biomedical Engineering, Evanston, 60208, USA; 2 Northwestern University, Department of Mechanical Engineering, Evanston, 60208, USA

## Abstract

Optical imaging has offered unique advantages in material researches, such as spectroscopy and lifetime measurements of deeply embedded materials, which cannot be matched using electron or scanning-probe microscopy. Unfortunately, conventional optical imaging cannot provide the spatial resolutions necessary for many nanoscopic studies. Despite recent rapid progress, super-resolution optical imaging has yet to be widely applied to non-biological materials. Herein we describe a method for nanoscopic optical imaging of buried polymer nanostructures without the need for extrinsic staining. We observed intrinsic stochastic fluorescence emission or blinking from unstained polymers and performed spatial-temporal spectral analysis to investigate its origin. We further applied photon localization super-resolution imaging reconstruction to the detected stochastic blinking, and achieved a spatial resolution of at least 100 nm, which corresponds to a six-fold increase over the optical diffraction limit. This work demonstrates the potential for studying the static heterogeneities of intrinsic polymer molecular-specific properties at sub-diffraction-limited optical resolutions.

Over the last two decades, nanostructured polymers have contributed to critical advances in both material and biological researches^1^. Down-scaling polymer structures to nanoscopic dimensions dramatically may alter their physical properties and thus bring new applications. Considering recent advances in nanofabrication and its application to polymeric materials^2^,^3^,^4^,^5^,^6^,^7^,^8^, new imaging methods are necessary to characterize both the geometrical and physical properties of nanoscopic polymer structures. Despite the success of electron microscopy (EM) and scanning probe microscopy (SPM), they are insufficient for non-destructive imaging of internal polymer structures beyond the superficial layer. In contrast, optical microscopy can non-destructively image embedded internal features at depths beyond that of EM or SPM. For example, optical microscopy can monitor internal single molecule distributions and locate defects inside crystals^9^,^10^,^11^. However, the spatial resolution of conventional optical imaging methods is fundamentally limited by the optical diffraction limit, which is far worse than the spatial resolution of EM and SPM. Therefore, developing super-resolution optical imaging methods using only a materials’ intrinsic physical or chemical properties offers unique advantages in the visualization and characterization of subsurface polymeric structures.

Recent advances in super-resolution optical imaging have significantly narrowed the resolution gap between optical imaging and EM or SPM techniques. Super-resolution optical imaging techniques, such as stochastic optical reconstruction microscopy (STORM)^12^, photoactivated localization microscopy (PALM)^13^, stimulated emission depletion (STED)^14^,^15^,^16^, and structured illumination microscopy (SIM)^17^,^18^,^19^, extend the ability to study sub-diffraction-limited features that were previously thought to be unresolvable by optical microscopy^20^. These technologies can reach spatial resolutions higher than 10 nm. Moreover, multi-contrast 3D optical super-resolution has been achieved with axial resolutions less than 40 nm^21^,^22^,^23^.

Previous studies primarily harnessed super-resolution for biological imaging and overlooked some unique applications in material studies. For example, material studies that utilized super-resolution techniques have successfully mapped polymer proton distribution^24^, mapped emitting sites within single conjugated polymer^25^, added tools for optimizing lithography techniques^24^, increased fundamental insight on hot-spot formation in nanostructures^26^, directly observed catalytic effects of metallic nanoparticles on a molecular scale^27^, and tracked single polymer molecules^28^. The vast majority of optical super-resolution technologies rely on extrinsic contrast agents. Extrinsic agents have several weaknesses, including (1) they require additional labeling processes; (2) they may modify the physical properties of the target material; and (3) they could introduce inaccurate spatial localization caused by the physical dimension of the tagged fluorescent and linker molecules. The combination of these weaknesses reduces the appeal of extrinsic fluorescent contrast agents. In fact, topological and chemical defects in polymeric molecules can result in various photophysical interactions, including energy transfer^29^,^30^,^31^, ground- or excited-state aggregate formation^32^,^33^, and charge transfer^34^. These photophysical processes can significantly modify some of the polymer’s material and optical properties^28^,^29^,^35^ that may be suitable for optical super-resolution imaging without the need of extrinsic labeling.

Here we report our investigation of stochastic switching of intrinsic fluorescence from polymers outside of the commonly recognized absorption-emission bands. Then we subsequently conduct photon-localization optical super-resolution imaging on the detected stochastic switching from unstained polymer nanostructures. We recently observed stochastic switching of molecular fluorescence (blinking) within the visible spectrum from several polymers (see Supplementary Fig. S1), including polymethyl methacrylate (PMMA), poly-styrene (PS), and SU-8 (SU-8 2005, Microchem), under modest illumination beam fluence (1–10 kW/cm^2^) in a wide-field illumination scheme (see Supplementary Fig. S2). The reported absorption band of PMMA and PS is less than 350 nm^36^,^37^, while the main absorption peak of SU-8 is also reported to be less than 350 nm with lower absorption in the visible range^38^. However, our results indicate that these polymers exhibit sparse blinking in the visible spectral range when excited by a 532-nm illumination beam. The presence of the blinking from long-chain polymers was confirmed with polymer thin films coated on a glass substrate. Spectroscopic and temporal analyses of the detected blinking events showed two characteristic stages. We also investigated blinking frequency (number of blinking events per second) and blinking photon count (the number of photons detected in a single blinking event) to verify the feasibility of STORM. We further determined the influence of surface coating (water immersion) on blinking frequency characteristics. Lastly, we used the intrinsic blinking to reconstruct the sub-diffraction-limited features of patterned PMMA nanostructures and achieved a six-fold improvement over the corresponding theoretical diffraction limit.

## Results

**Figure 1:**
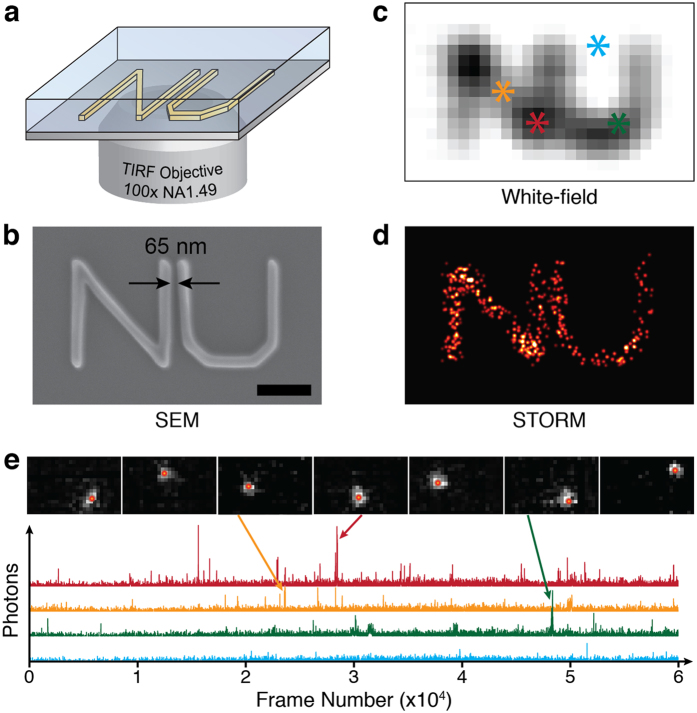
(**a**) 3D rendering of the fabricated NU pattern on glass substrate imbedded in a material. Imbedding the film in material makes SEM imaging difficult. (**b**) SEM image of the PMMA nanopatterned target on glass; the scale bar is 1 μm. To show the diffraction limit, wide-field white-light imaging was performed on the NU target (**c**). As expected, the pattern could not be resolved. The background is removed to make the location of the PMMA clear. On the other hand, the STORM reconstructed image (**d**) clearly shows the NU nanopattern to resolutions far greater than the diffraction limit. Representative blinking events (**e**) were selected from the NU pattern reconstruction video for reader understanding. The central position calculated by the localization algorithm is displayed in each consecutive image. The inset of (**e**) shows the detector radiation from 60,000 frames. The color-coded asterisks, shown in (**b**), represent the spot location of each radiation photon count data set shown.

As shown in Fig. 1a, we used PMMA to create nanopatterns on a glass substrate for use in initial blinking tests and STORM imaging. Figure 1b shows a scanning electron microscopy (SEM) image of an “NU” pattern fabricated using E-Beam lithography (see Supplementary Information for fabrication details) with a 100 nm linewidth in a 200 nm-thick PMMA film. The 65-nm gap between the letters (highlighted in Fig. 1b) cannot be resolved using wide-field optical microscopy (Fig. 1c) because the feature size is below the diffraction-limited resolution (225 nm) using a 1.49-NA TIRF objective (Supplement Fig. S2). A 532-nm laser was used to illuminate the sample and 60,000 frames were recorded with integration time of 33-ms per frame at a frame rate of 30 Hz (see detailed imaging procedure in Methods). Reconstruction was conducted using the localized stochastic blinking based on STORM principles (Fig. 1d). We monitored multiple locations of the glass substrate, with and without PMMA coating, for blinking events. We were able to observe stochastic blinking events from the “NU” pattern (Fig. 1e) under modest illumination beam fluences (5 kW/cm^2^) at an average blinking frequency of 0.72 (events/second) per μm^2^ (see Supplementary Movie 1).

**Figure 2:**
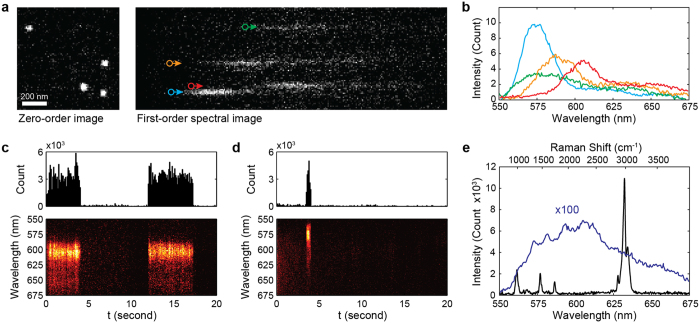
(**a**) A representative frame collected by the EMCCD for spectroscopy. Image was separated according to the diffraction order of the grating. (**b**) Fluorescence spectra of individual blinking events denoted by colored circles in (**a**). (**c**) Shows the emission from long-lived blinking events at a single location and the respective spectra in time. The spectra and photon count were consistent for each consecutive flashing event. Blinking from short-lived events (**d**) also had consistent spectra for each location, however, they were comparatively narrower and blue-shifted in PMMA. For longer integration times, Raman background was more dominant than blinking event intensities (**e**). Therefore, to obtain accurate blinking emission spectra, the stochastic emission was summed over 1,000 consecutive frames and multiplied by 100 for comparison.

To understand what type of optical radiation generated the observed blinking events, we conducted spectroscopy experiments on polymer thin films (see experimental setup in Supplementary Fig. S2). We added a secondary light path for fluorescence spectroscopy in our custom-built STORM microscope. We excited the 200-nm thick PMMA film samples with a 532-nm laser at illumination beam fluences ranging from 1–10 kW/cm^2^. We acquired the spectral information of each individual blinking event using a high-speed electron-multiplied CCD (EMCCD) (ProEM512, Princeton) attached to a spectrometer (SP2150i, Princeton) with a 0.6 nm maximum spectral resolution. Since an individual blinking event can be treated as a sub-diffraction-limited point source, the high resolution spectrum can be captured by the monochromator without the need for an entrance slit (see Fig. 2a). The measured zero-order image (left panel in Fig. 2a) not only determines the precise location of each blinking event, it also establishes the reference point to identify the associated emission spectrum measured from the first-order image (Fig. 2b). The detailed information can be found in the Supplementary Information.

Upon laser beam illumination of the PMMA thin film, we observed rapid temporal decay of the spontaneous fluorescent radiation within 60 seconds followed by a stabilized blinking stage that lasted more than 60 minutes. The observed temporal decay of fluorescent radiation is likely caused by the non-irreversible photophysical bleaching process^39^. The STORM experiment was performed when the sample reached the stabilized blinking stage. We monitored the duration of the stochastic blinking in the PMMA thin film by acquiring 200 consecutive images using a detector integration time of 100 ms per frame (also see results of other polymers in Supplementary Fig. S3). Figure 2c shows the blinking photon counts and associated spectra from detected “long-lived” blinking events, which lasted up to 15 seconds. Figure 2d shows the blinking photon counts and associated spectra from detected “short-lived” blinking events, which usually lasted for tens of milliseconds. In addition, as shown in Fig. 2c,d, we found that individual blinking events have comparable blinking photon counts regardless of the location in the sample, but the spectra are highly dependent on the locations. However, localized events, likely from the same molecule or potentially sub-molecular structure of the long-chain polymeric molecule, displayed nearly identical spectral information regardless of the frequency and duration of the blinking events. Spectra of “short-lived” blinking events were characteristically blue-shifted and had narrower spectra compared with “long-lived” blinking events. Since all the observed stochastic blinking events were temporally sparse, we identified an optimal detector integration time to avoid a strong accumulated PMMA Raman scattering background. To obtain accurate spectra without the influence from Raman scattering, we acquired high-speed video using the EMCCD detector and subsequently removed the constant Raman scattering background. Using high imaging speed (85 fps), the Raman scattering background was less obvious compared with the observed stochastic blinking (see details of measurement in the Supplementary Information). To determine the spectral characteristics of these blinking events, we averaged the optical spectrum of all the blinking events detected within a 10-minute period (Fig. 2e). Unlike the PMMA Raman scattering spectrum (also shown in Fig. 2e), the averaged spectrum from all blinking events has a broad spectral range suggesting that it is from fluorescence.

**Figure 3:**
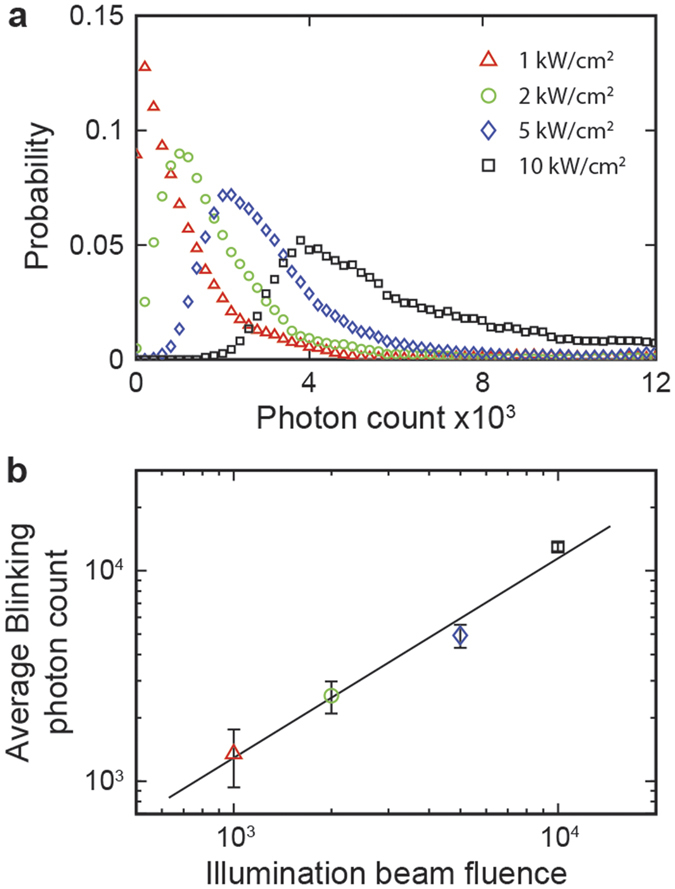
Increases in illumination beam fluence resulted in blinking with higher photon counts (**a**) and an overall linear increase in averaged emission (**b**). The linear curve fitting of (**b**) revealed a slope of approximately 1 (0.95), experimentally verifying the linear relation between illumination beam fluence and photon emission.

Intrinsic fluorescent blinking in polymers has been well studied^29^,^30^,^31^,^32^,^33^,^34^; however, blinking from PMMA, PS, and SU-8 has not previously been reported. To confirm if the observed blinking was environmentally or photophysically induced, we varied the illuminating beam fluence (1, 2, 5 and 10 kW/cm^2^) and measured the corresponding changes in blinking frequency and blinking photon count. Photophysically induced changes have a linear dependence on the illuminating beam fluence, while environmentally induced changes are beam-fluence-independent^29^. Figure 3a shows how the illumination beam fluence affects the probability density function of the blinking photon counts. When the illumination beam fluence increased the probability of blinking events with larger photon count also increased. Figure 3b shows the relationship between the blinking photon count and the illumination beam fluence in a log-log plot. Our least-square fit suggested a linear slop of 0.95, showing that linear increases in illumination beam fluence resulted in a corresponding linear increase in photon count from individual blinking. This experiment confirms that all the fluorescent blinking events are the results of photophysical changes induced by laser beam illumination.

**Figure 4:**
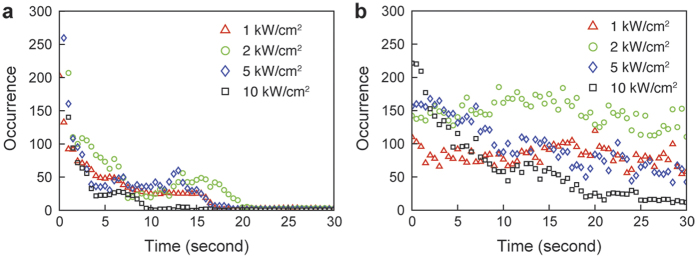
Occurrences of fluorescence emission with respect to beam illumination duration was measured at different illumination beam fluences from (**a**) air and (**b**) water-immersed PMMA films. We continuously measured the number of total fluorescence emission events within each 100-ms integration time for 30 seconds. The occurrence from the air sample dropped significantly faster than from the water-immersed sample. The initial decay stage lasts for about 30 seconds, but is most prominent in the first 5 seconds. We then observed stable blinking at an average frequency of 0.013 events per second at 2 kW/cm^2^ and 0.582 events per second at 2 kW/cm^2^ per square micrometer from the air and water-immersed PMMA film samples, respectively.

We then investigated temporal characteristics of the observed fluorescence emission, including the temporal decaying stage and the stabilized blinking stage, under different illumination beam fluences as shown in Fig. 4. We used a frame rate of 100 fps to capture the changes in blinking frequency in both stages. In the temporal decaying stage, we counted the occurrences of fluorescence emission within the first 30 seconds. Then, we continued the illumination for 5 minutes under the same fluence, after which we measured the stabilized blinking frequency for up to one hour. For PMMA film samples tested in air, rapid temporal decaying could be observed for less than 20 seconds at all the tested illumination beam fluences (Fig. 4a). As expected, the temporal decaying stage had the shortest decay time under the highest illumination beam fluence.

We also investigated how water immersion affects blinking frequency under the same illumination beam fluences as the aforementioned PMMA sample in air. Photo-oxidation of the polymer matrix reduced photo-recovery (the ability of the emitting molecule(s) to make multiple transitions to on-off fluorescent states, allowing for multiple blinking events) of the blinking sites^40^,^41^, which could change the blinking frequency. We immersed the PMMA film in deionized-water to potentially reduce the oxidation effects and observed that water-immersed PMMA films showed a slower temporal decay (Fig. 4b) than that of dry PMMA films (Fig. 4a). The average photon count from all individual blinking events was comparable for both dry and water-immersed PMMA. Furthermore, in both samples, the blinking frequencies and photon counts stabilized after exposure for several minutes, but the blinking frequency was significantly lower in the dry PMMA than the water-immersed PMMA. Therefore, we conclude that oxidation is less likely in water-immersed samples, allowing more stable photo-emission (intrinsic fluorescence).

Finally, we determined the spatial resolution of our STORM based on intrinsic blinking contrast both theoretically and experimentally using fabricated PMMA samples. Depending on detector parameters and filter efficiency, the theoretical resolution of STORM imaging can vary. If the probability of simultaneous stochastic light generation from multiple nearby regions is negligible (sparsity requirement), we can assume the detected point-spread-function, or PSF, is from a single stochastic event. The center of the PSF can be approximated by the probability equation,


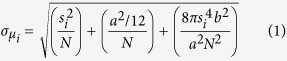


where *N* is the number of detected photons; *S*_*i*_ is the standard deviation of the intensities of the PSF; *a* is the detector pixel size; and *b* is the standard deviation of the background noise. As the number of detected photons determines the probabilistic center of the PSF, resolution is limited by the photon counts of the stochastic blinking, the detector background, and the efficiency of the optical setup. Using the probability equation and considering the background of the detector, our experimental resolution was calculated to be approximately 37 nm for the “NU” nanopatterned sample.

**Figure 5:**
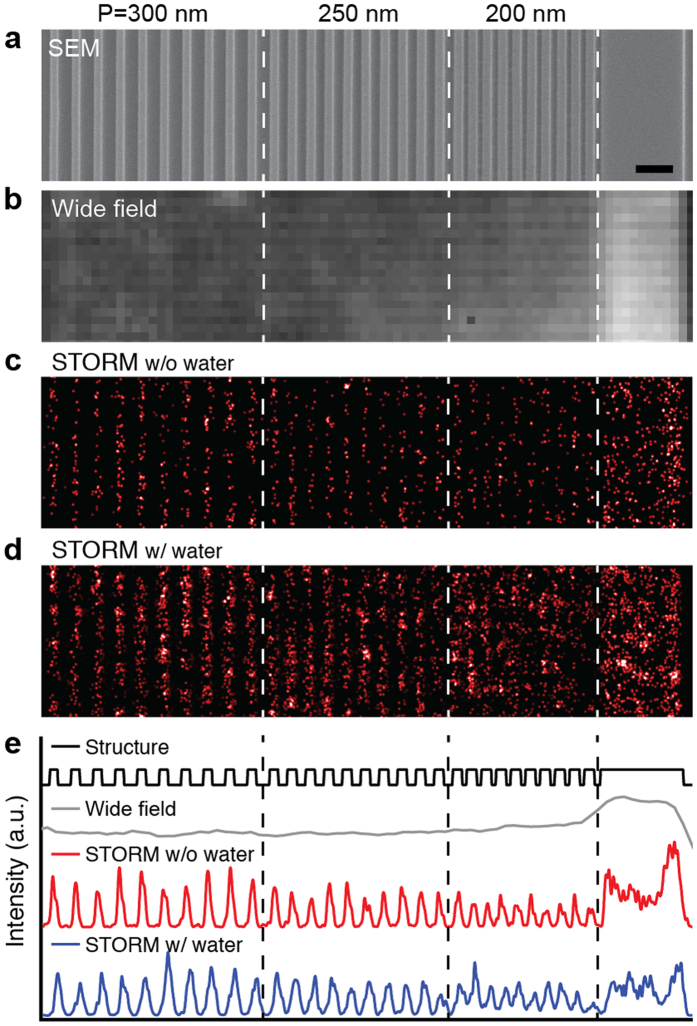
(**a**) SEM of the grated PMMA target nanopattern was used to confirm sample periodicity. White-light wide-field imaging (**b**) was attempted but, as expected, could not resolve the diffraction limited features; however, intrinsic STORM of both dry (**c**) and water-immersed (**d**) samples successfully resolved the diffraction limited features. The dry sample had significantly fewer events, resulting in a poorly reconstructed patter; however, resolution of both (**c,d**) is equivalent due to the similar photon count of stochastic events in dry and immersed samples. To show the periodicity more clearly, the 2D structure intensity was integrated vertically to show the 1D intensity histogram (**e**). The scale bar represents 500 nm.

To experimentally quantify the spatial resolution, we patterned a PMMA sample with progressively increasing grid periodicities (200 nm, 250 nm, 300 nm and 400 nm) for resolution tests (Fig. 5). We confirmed the periodicity and gap spacing of the PMMA grid sample using SEM (Fig. 5a). As expected, conventional wide-field imaging (Fig. 5b) did not resolve the line spacing of the PMMA grid sample. To quantify our STORM resolution, we collected 60,000 frames for image reconstruction. The reconstructed image from the PMMA grid sample in air is shown in Fig. 5c and the image from a water-immersed PMMA sample is shown in Fig. 5d. We further averaged the pixels along the vertical axis to create line profiles of the intensity distributions as shown in Fig. 5e. Clearly, patterns with periodicity of 200 nm can be well resolved for both air and water-immersed PMMA samples, indicating a half-pitch resolution better than 100 nm. For the water-immersed PMMA sample, a clearer image of the nanopattern was reconstructed due to increased blinking frequency. The actual system resolution could be higher than the spacing of the nanopatterned sample. Therefore, using the edge-spread-function measured from the large PMMA rectangle (5 μm × 1 μm) with a sharp edge, we determined our STORM resolution to be 45 nm (Supplementary Fig. S4). The experimentally quantified resolution is lower than the theoretical estimate due to perturbations, such as fluctuations in sample position from stage drift.

## Discussion

The reported absorption bands of PMMA and other tested lithographic polymers are usually in the UV spectral regime^36^,^37^,^38^. As previously reported^29^,^30^,^42^,^43^,^44^,^45^, the intramolecular energy transfer, defect sites, microscopic molecular restructuring, or peroxidation can affect the polymers absorption band, allowing polymer molecules being possibly excited outside their main absorption bands. Since we observed blinking from uncured samples, in addition to E-Beam treated samples, we think that electron bombardment from E-Beam lithography is unlikely to be the solo cause of blinking. Blinking in polymers can also be attributed to impurity luminescence in polymer samples^35^. However, in our investigation, blinking mainly occurred at the polymer surface, whereas little blinking was observed inside thicker bulk films. Therefore, it is unlikely that native impurities alone are responsible for the blinking. In addition, we observed an increase in blinking frequency at intentionally damaged (scratched) sites on PMMA films. Damaged sites exhibited altered Raman peak ratios for the stretching vibrations of the C-H methylene group bands (see Supplementary Fig. S5), which indicate conformational state changes in the polymer structure may be associated with blinking. Therefore, such blinking is likely to come from intrinsic fluorescence from PMMA or PS polymers. It should also be noted that PMMA and PS are commonly used to immobilize and study optical properties from other molecules embedded in their polymer matrix. Illumination beam fluences reported in literature^46^,^47^ to excite non-native molecules embedded in the polymer matrix typically range from 8 W/cm^2^–3 kW/cm^2^, which is within the illumination fluence range we used for intrinsic blinking observation (1–10 kW/cm^2^). It is possible that, if intrinsic blinking of host polymers is not considered, investigations of the optical properties of non-native embedded molecules can be affected.

While stochastic switching of single-molecule fluorescence has been broadly reported, this phenomenon has not been investigated in many commonly used polymers. Particularly in this study, intensive blinking events were observed in PMMA, PS, and SU-8 films. Such intrinsic blinking can possibly be explained by intensity jumps from intramolecular energy transfer to molecular chain defect sites^29^ or small changes in polymer microscopic structure^30^,^44^,^45^; thus affecting absorption band characteristics. In both cases, statics changes affect spectral emission^44^, causing different emission spectra from different blinking locations, but consistent spectra for each individual blinking location. Therefore, STORM allows direct study of the static heterogeneities of each individual molecule instead of the average properties of the whole sample.

Using intrinsic blinking from widely used polymers for high-resolution imaging provides an advantage over EM and SPM. For example, imaging polymers with EM commonly causes structure damage from electron bombardment or requires heavy metal staining. Also, studies that create nanoscopic structures to be used in liquid environments cannot be imaged simultaneously with electron microscopy. For instance, the properties of the previously mentioned immersed PMMA nanostructure cannot be used in the clean vacuum environments necessary for EM. In addition, optically transparent polymers are opaque in EM and SPM, eliminating the possibility to nondestructively study deeply embedded materials (one of the common uses of lithographic polymers). Furthermore, single-molecule dynamics are more easily studied using super-resolution optical microscopy. As a result, super-resolution optical techniques can provide technological advantages as compared with EM or SPM.

In this work, we demonstrated the optical nanoscopic imaging of buried polymer samples using intrinsic fluorescence. We confirmed the fluorescent characteristics of PMMA thin films using fluorescence spectroscopy and further confirmed the photophysical origin of the blinking by adjusting illumination beam fluence and measuring the corresponding blinking properties. We performed statistical investigation of blinking frequency and photon count to verify the feasibility of STORM imaging. To understand the environmental factors related to the observed blinking process, we modified the surface environment by covering the PMMA thin film with deionized-water. We compared the water-immersed PMMA film properties with those of non-immersed film using STORM.

STORM offers nanoscopic resolutions using simple wide-field illumination. Based on the results shown above, our technique is especially suited for studies involving embedded polymers with intrinsic blinking. Hence, our method may provide a new tool to study, for example, individual molecule static heterogeneities in polymer structures^1^ and to develop new polymer-based STORM contrast agents.

## Methods

### STORM and fluorescence spectroscopy setup

We built an integrated optical imaging and spectroscopy system based on an inverted microscope as shown in Fig. S1. A 532-nm monochromatic laser beam (Lambdapro Technologies, UG-120 mW DPSS) was passed through the microscope body (Nikon, Eclipse Ti-U) and was focused by an objective lens (Nikon, TIRF 100X, 1.49NA). The intensity and beam size of the illumination beam fluence were controlled by a linear polarizer and a dual lens assembly. For spectral characterization, the signal was routed to a spectrometer (Princeton, SP2150i) with a 150 lines/mm diffraction grating and an EMCCD (Princeton Instruments, ProEM512B Excelon), giving a maximum 0.6-nm spectral resolution. The primary image was collected through a 550-nm long-pass filter before video acquisition by an EMCCD (Andor, iXon 897 Ultra). Imaging and spectral experiments were performed in multiple polymers, including PMMA, PS, and SU-8.

### Sample preparation

Polymer thin films for spectroscopic study were prepared on glass substrates using spin-coating method at 4000 rpm for 45 seconds. The thicknesses of the PMMA and PS films are 200 nm and the thickness of the SU-8 films is 800 nm. We further baked the film samples on a hot plate at 180 °C for 1 minute to evaporate extra solvent.

PMMA nanostructure samples were created on glass substrate using E-Beam lithography nanopatterning. A 200 nm-thick polymethyl methacrylate (950PMMA, MicroChem) layer was spin-coated (Laurell WS-650-23) on an indium-tin-oxide coated glass cover slip at 4000 rpm for 45 seconds. It was further baked on a hot plate at 180 °C for 1 minute to evaporate extra solvent. After patterning with an E-beam lithography system (FEI Quanta 600F) as a high-resolution negative resist, the unexposed PMMA was dissolved in acetone for 1 minute and then cleaned with distilled water and air-dried. We patterned several structures, including “NU” logo with an approximate 100-nm gap between letters and PMMA grid patterns with periodicity of 200, 250, 300 and 400 nm.

### Imaging procedure

Samples were placed on the microscope stage and imaged using a high-NA TIRF objective. We used a 532-nm laser with constant beam fluence of 5 kW/cm^2^ to illuminate the sample. After exposing under laser beam illumination for at least 5 minutes, we started to record images in the stabilized blinking stage using the EMCCD camera. The integration time and the frame rate of image acquisition was carefully selected to provide optimal signal-to-noise ratio of the acquired image. Unless specifically noted, 60,000 frames were recorded for STORM reconstruction.

### Experimental quantification of the lateral resolution

We were able to observe 65 nm spacing between the letters of the PMMA “NU” pattern (Fig. 1). However, the actual experimental resolution could not be directly determined using either the “NU” or PMMA grating nanostructures. Instead, we measured the edge-spread-function from a 200-nm-thick solid PMMA rectangle with a width of 1 μm and length of 5 μm (Fig. S3). We obtained the line-spread-function from the edge-spread-function and estimated the spatial resolution as the full-width-half-maximum value of the line-spread-function.

## Additional Information

**How to cite this article**: Urban, B. E. *et al*. Subsurface Super-resolution Imaging of Unstained Polymer Nanostructures. *Sci. Rep.* **6**, 28156; doi: 10.1038/srep28156 (2016).

## Supplementary Material

Supplementary Information

Supplementary Movie 1

## References

[b1] Wöll D. . Polymers and single molecule fluorescence spectroscopy, what can we learn? Chemical Society Reviews 38, 313–328 (2009). 19169450 10.1039/b704319h

[b2] Gates B. D. . New approaches to nanofabrication: molding, printing, and other techniques. Chemical reviews 105, 1171–1196 (2005). 15826012 10.1021/cr030076o

[b3] Lu Y. & Chen S. Micro and nano-fabrication of biodegradable polymers for drug delivery. Advanced drug delivery reviews 56, 1621–1633 (2004). 15350292 10.1016/j.addr.2004.05.002

[b4] Wei G. & Ma P. X. Structure and properties of nano-hydroxyapatite/polymer composite scaffolds for bone tissue engineering. Biomaterials 25, 4749–4757 (2004). 15120521 10.1016/j.biomaterials.2003.12.005

[b5] Liu X. & Wang S. Three-dimensional nano-biointerface as a new platform for guiding cell fate. Chemical Society Reviews 43, 2385–2401 (2014). 24504119 10.1039/c3cs60419e

[b6] Cui Z., Drioli E. & Lee Y. M. Recent progress in fluoropolymers for membranes. Progress in Polymer Science 39, 164–198 (2014).

[b7] Jin L., Zeng X., Liu M., Deng Y. & He N. Current progress in gene delivery technology based on chemical methods and nano-carriers. Theranostics 4, 240 (2014). 24505233 10.7150/thno.6914 PMC3915088

[b8] Chan Y. H. & Wu P. J. Semiconducting polymer nanoparticles as fluorescent probes for biological imaging and sensing. Particle & Particle Systems Characterization 32, 11–28 (2015).

[b9] Endesfelder U. Advances in Correlative Single-Molecule Localization Microscopy and Electron Microscopy. NanoBioImaging 1 (2014).

[b10] Kim S. . All-water-based electron-beam lithography using silk as a resist. Nature nanotechnology 9, 306–310 (2014). 10.1038/nnano.2014.47 24658173

[b11] Arroyo-Camejo S. . Stimulated emission depletion microscopy resolves individual nitrogen vacancy centers in diamond nanocrystals. ACS nano 7, 10912–10919 (2013). 24245613 10.1021/nn404421b

[b12] Rust M. J., Bates M. & Zhuang X. Stochastic optical reconstruction microscopy (STORM) provides sub-diffraction-limit image resolution. Nature methods 3, 793 (2006). 16896339 10.1038/nmeth929 PMC2700296

[b13] Hess S. T., Girirajan T. P. & Mason M. D. Ultra-high resolution imaging by fluorescence photoactivation localization microscopy. Biophysical journal 91, 4258–4272 (2006). 16980368 10.1529/biophysj.106.091116 PMC1635685

[b14] Hell S. W. & Wichmann J. Breaking the diffraction resolution limit by stimulated emission: stimulated-emission-depletion fluorescence microscopy. Optics letters 19, 780–782 (1994). 19844443 10.1364/ol.19.000780

[b15] Hell S. W. Far-field optical nanoscopy. Science 316, 1153–1158 (2007). 17525330 10.1126/science.1137395

[b16] Klar T. A., Jakobs S., Dyba M., Egner A. & Hell S. W. Fluorescence microscopy with diffraction resolution barrier broken by stimulated emission. Proceedings of the National Academy of Sciences 97, 8206–8210 (2000). 10.1073/pnas.97.15.8206 PMC26924 10899992

[b17] Gustafsson M. G. Surpassing the lateral resolution limit by a factor of two using structured illumination microscopy. Journal of Microscopy 198, 82–87 (2000). 10810003 10.1046/j.1365-2818.2000.00710.x

[b18] Gustafsson M. G. Nonlinear structured-illumination microscopy: wide-field fluorescence imaging with theoretically unlimited resolution. Proceedings of the National Academy of Sciences of the United States of America 102, 13081–13086, 10.1073/pnas.0406877102 (2005). 16141335 PMC1201569

[b19] Urban B. E. . Super-resolution two-photon microscopy via scanning patterned illumination. Physical Review E 91, 042703 (2015). 10.1103/PhysRevE.91.042703 PMC4565794 25974523

[b20] Gordon J. An Examination of the Abbe Diffraction Theory of the Microscope. Journal of the Royal Microscopical Society 21, 353–396 (1901).

[b21] Schmidt R. . Spherical nanosized focal spot unravels the interior of cells. Nature Methods 5, 539–544 (2008). 18488034 10.1038/nmeth.1214

[b22] Huang B., Jones S. A., Brandenburg B. & Zhuang X. Whole-cell 3D STORM reveals interactions between cellular structures with nanometer-scale resolution. Nature Methods 5, 1047–1052 (2008). 19029906 10.1038/nmeth.1274 PMC2596623

[b23] Huang B., Wang W., Bates M. & Zhuang X. Three-dimensional super-resolution imaging by stochastic optical reconstruction microscopy. Science 319, 810–813 (2008). 18174397 10.1126/science.1153529 PMC2633023

[b24] Berro A. J., Berglund A. J., Carmichael P. T., Kim J. S. & Liddle J. A. Super-resolution optical measurement of nanoscale photoacid distribution in lithographic materials. ACS nano 6, 9496–9502 (2012). 23102414 10.1021/nn304285m

[b25] Habuchi S., Onda S. & Vacha M. Mapping the emitting sites within a single conjugated polymer molecule. Chemical Communications, 4868–4870 (2009). 19652808 10.1039/b907882g

[b26] Willets K. A., Stranahan S. M. & Weber M. L. Shedding light on surface-enhanced Raman scattering hot spots through single-molecule super-resolution imaging. The Journal of Physical Chemistry Letters 3, 1286–1294 (2012). 26286772 10.1021/jz300110x

[b27] Zhou X. . Quantitative super-resolution imaging uncovers reactivity patterns on single nanocatalysts. Nature nanotechnology 7, 237–241 (2012). 10.1038/nnano.2012.18 22343380

[b28] King J. T., Yu C., Wilson W. L. & Granick S. Super-resolution study of polymer mobility fluctuations near c*. ACS nano 8, 8802–8809 (2014). 25148053 10.1021/nn502856t

[b29] Bout D. A. V. . Discrete intensity jumps and intramolecular electronic energy transfer in the spectroscopy of single conjugated polymer molecules. Science 277, 1074–1077 (1997).

[b30] Yu J., Hu D. & Barbara P. F. Unmasking electronic energy transfer of conjugated polymers by suppression of O_2_ quenching. Science 289, 1327–1330 (2000). 10958774 10.1126/science.289.5483.1327

[b31] Brédas J.-L., Beljonne D., Coropceanu V. & Cornil J. Charge-transfer and energy-transfer processes in π-conjugated oligomers and polymers: a molecular picture. Chemical Reviews 104, 4971–5004 (2004). 15535639 10.1021/cr040084k

[b32] Romaner L. . The Origin of Green Emission in Polyfluorene-Based Conjugated Polymers: On-Chain Defect Fluorescence. Advanced Functional Materials 13, 597–601 (2003).

[b33] Nguyen T.-Q., Doan V. & Schwartz B. J. Conjugated polymer aggregates in solution: Control of interchain interactions. The Journal of Chemical Physics 110, 4068–4078 (1999).

[b34] Noone K. M. . Photoinduced charge transfer and polaron dynamics in polymer and hybrid photovoltaic thin films: organic vs inorganic acceptors. The Journal of Physical Chemistry C 115, 24403–24410 (2011).

[b35] Tian Y. . Molecular Weight Determination by Counting Molecules. The Journal of Physical Chemistry Letters 6, 923–927 (2015). 26262846 10.1021/acs.jpclett.5b00296

[b36] Coustet M. E. & Cortizo M. S. Functionalization of styrenic polymer through acylation and grafting under microwave energy. Polymer Journal 43, 265–271 (2011).

[b37] Monika C., Vazid A. & Sushil K. Spectral Investigations of Kiton Red-620 Doped Polymethylmethacrylate. Materials Sciences and Applications 2012 (2012).

[b38] Parida O. P. & Bhat N. Characterization of optical properties of SU-8 and fabrication of optical componenets. Paper presented at *ICOP Proceedings of the International Conference on Optics and Photonics, Chandigarh, India.* Chandigarh: CSIO. (2009, October 30-November 1).

[b39] Ha T. & Tinnefeld P. Photophysics of fluorescence probes for single molecule biophysics and super-resolution imaging. Annual Review of Physical Chemistry 63, 595 (2012). 10.1146/annurev-physchem-032210-103340 PMC3736144 22404588

[b40] Park S.-J., Gesquiere A. J., Yu J. & Barbara P. F. Charge injection and photooxidation of single conjugated polymer molecules. Journal of the American Chemical Society 126, 4116–4117 (2004). 15053595 10.1021/ja031929x

[b41] Cunliffe A. & Davis A. Photo-oxidation of thick polymer samples—Part II: The influence of oxygen diffusion on the natural and artificial weathering of polyolefins. Polymer Degradation and Stability 4, 17–37 (1982).

[b42] Deepak K. L. N., Desai N. R. & Soma V. R. Direct Writing in Polymers with Femtosecond Laser Pulses: Physics and Applications. (INTECH Open Access Publisher, 2012).

[b43] Nurmukhametov R., Volkova L. & Kabanov S. Fluorescence and absorption of polystyrene exposed to UV laser radiation. Journal of Applied Spectroscopy 73, 55–60 (2006).

[b44] Ebihara Y. & Vacha M. Relating conformation and photophysics in single MEH-PPV chains. The Journal of Physical Chemistry B 112, 12575–12578 (2008). 18793023 10.1021/jp806963u

[b45] Köhn F., Hofkens J., Gronheid R., Van der Auweraer M. & De Schryver F. C. Parameters influencing the on-and off-times in the fluorescence intensity traces of single cyanine dye molecules. The Journal of Physical Chemistry A 106, 4808–4814 (2002).

[b46] Métivier R., Nolde F., Müllen K. & Basché T. Electronic excitation energy transfer between two single molecules embedded in a polymer host. Physical Review Letters 98, 047802 (2007). 17358814 10.1103/PhysRevLett.98.047802

[b47] Vallée R. . On the role of electromagnetic boundary conditions in single molecule fluorescence lifetime studies of dyes embedded in thin films. Chemical Physics Letters 348, 161–167 (2001).

