# Supplementary material for: Subsurface Super-resolution Imaging of Unstained Polymer Nanostructures
Source: Sci Rep. 2016 Jun 29;6:28156. doi: 10.1038/srep28156 (PMC4926209; doi:10.1038/srep28156)
Supplement: Supplementary Information [file srep28156-s1.doc]

**Supplement Information:**

Subsurface Super-resolution Imaging of Unstained Polymer Nanostructures

*Ben E. Urban^1^, Biqin Dong^1,2^, Quyen Nguyen^1^,Vadim Backman^1^, Cheng Sun^2^, and Hao F. Zhang^1*^*

^1^Northwestern University, Department of Biomedical Engineering, Evanston, 60208, USA

^2^Northwestern University, Department of Mechanical Engineering, Evanston, 60208, USA

E-mail: [hfzhang@northwestern.edu](mailto:hfzhang@northwestern.edu)

1. **Details of Sample Preparation**

Polymer thin films for single molecule spectroscopic study were prepared on glass cover slip using spin-coating method. The thickness of PMMA, PS and SU-8 films are 200 nm, 200 nm and 800 nm, respectively. These polymer films were used for studying the stochastic blinking phenomena and performing statistical analysis (as shown in Fig. 2 and 3).

PMMA nanostructure samples were created on glass substrate using e-beam lithography nanopatterning. A 200 nm-thick polymethyl methacrylate (950PMMA, MicroChem) layer was spin-coated (Laurell WS-650-23) on an indium-tin-oxide coated glass cover slip at 4000 rpm for 45 seconds. It was further pre-baked on a hot plate at 180 °C for 1 min to evaporate extra solvent. After patterning with an electron beam lithography system (FEI Quanta 600F) as a high-resolution negative resist, the unexposed PMMA was dissolved in acetone for 1 min and then cleaned with distilled water and air-dried. Structures were composed of “NU” logo letters with an approximate 100 nm gap between letters, and PMMA bar patterns with bars spaced at distances of 100, 150, 200 and 300 nm.

1. **Imaging system and spectroscopy**

An integrated optical imaging and spectroscopy system was built in our lab based on an inverted microscope as shown in Fig. S2. A 532 nm monochromatic laser (Lambdapro Technologies, UG-120 mW DPSS) was passed through the microscope body (Nikon, Eclipse Ti-U) and focused with an objective lens (Nikon, TIRF 100X, 1.49NA). The intensity and beam size of the laser fluence were controlled by a linear polarizer and a dual lens beam expender. The primary image was collected through a 550 nm long-pass filter before video acquisition by an EMCCD (Andor, iXon 897 Ultra). For spectral characterization, the signal was routed to a monochromator (Princeton, SP2150i) with a 150 lines/mm diffraction grating and an EMCCD (Princeton Instruments, ProEM512B Excelon), giving a maximum 0.63 nm spectral resolution. A mirror was placed in the spectrometer to control the position of the zero-order image so both zero-order and first order images can be simultaneously acquired by the same EMCCD camera. Since stochastic blinking event can be treated as a sub-diffraction-limited point source, the high resolution spectrum can be captured using the monochromator without the need for entrance slit. The zero-order image was reversed due to the additional mirror, but else remained identical to images obtained using generic STORM (without the monochromator), providing an inherent reference of the measured spectrum for each blinking event. The centroid positions provided the location of each activated stochastic event and are further used to calibrate the spectral coordinates of the corresponding first-order image to reveal the emission spectrum.

We observed Raman scattering signals from both the substrate and polymers after the 550-nm long-pass filter. Though Raman scattering signals were useful in confirming the polymers’ molecular composition, the presence of Raman scattering background could affect the STORM reconstruction accuracy if not properly removed. In particular, the Raman scattering is considered as a constant flux of photons, while the observed blinking events carry a burst of photons over of short time duration. Thus, long integration time (> 100 ms) will unfavorably increase the Raman background signals that may potentially overpower the observed intensities of intrinsic blinking signals. In the experiment, the images were acquired at a frame rate of 100 fps to sufficiently reduce the Raman background. In addition, the Raman scattering background was obtained by averaging the Raman signals from frames without detected intrinsic blinking signals. We then subtracted the average Raman scattering background from each individual frame to further reduce the background signal.

1. **Experimental quantification of the lateral resolution**

We were able to observe 65 nm spacing between the letters of the PMMA “NU” pattern (Fig. 1). However, the actual experimental resolution could not be directly determined using either the “NU” or PMMA grating nanostructures. Instead, we measured the edge spread function (ESF) from a 200-nm-thick solid PMMA bar with a width of 1 μm and length of 5 μm (Fig. S4). We obtained the line spread function (LSF) from ESF and estimated the spatial resolution as the FWHM of the LSF.


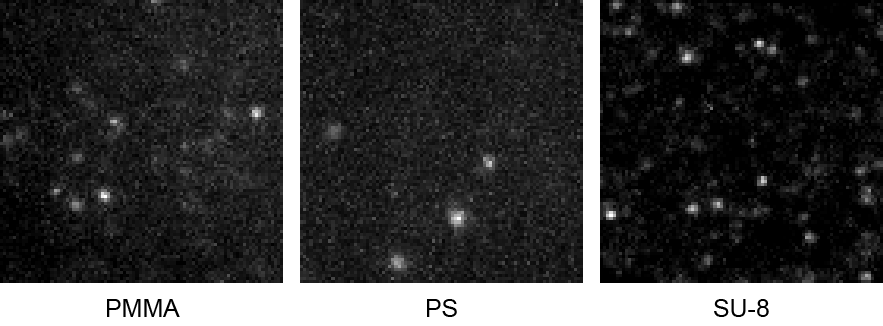


Fig. S1. Representative frames showing blinking events on PMMA, PS and SU-8 films.


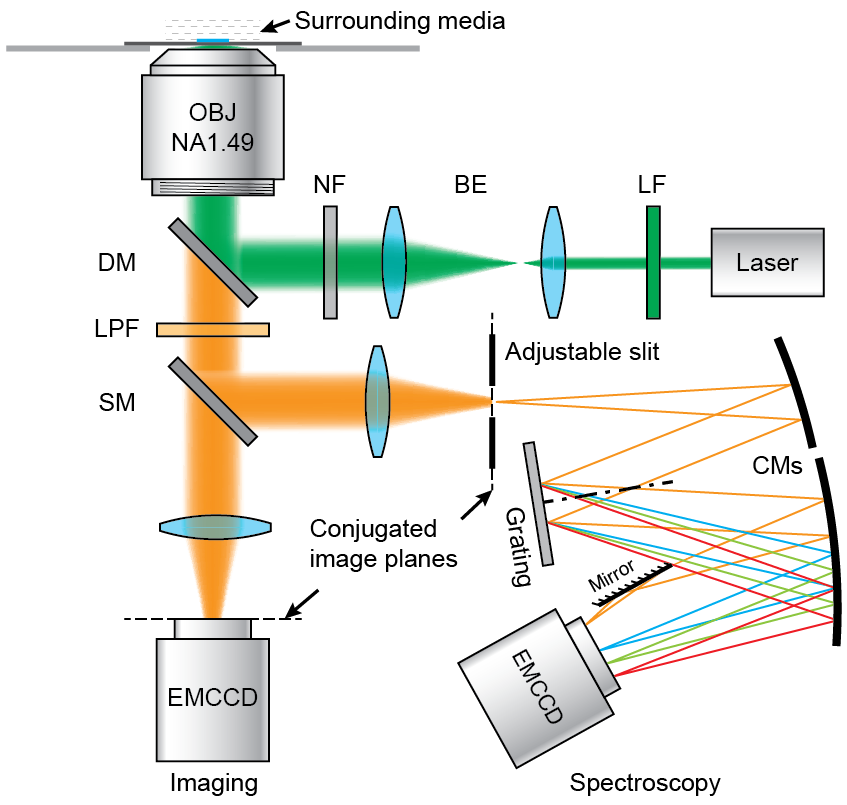


Fig. S2. Schematic of optical setup. LF: laser line filter, BE: beam expender, NF: Notch filter, DM: dichroic mirror, LPF: long pass filter, SM: switchable mirror, BF: band-pass filter, CM: concave mirror.


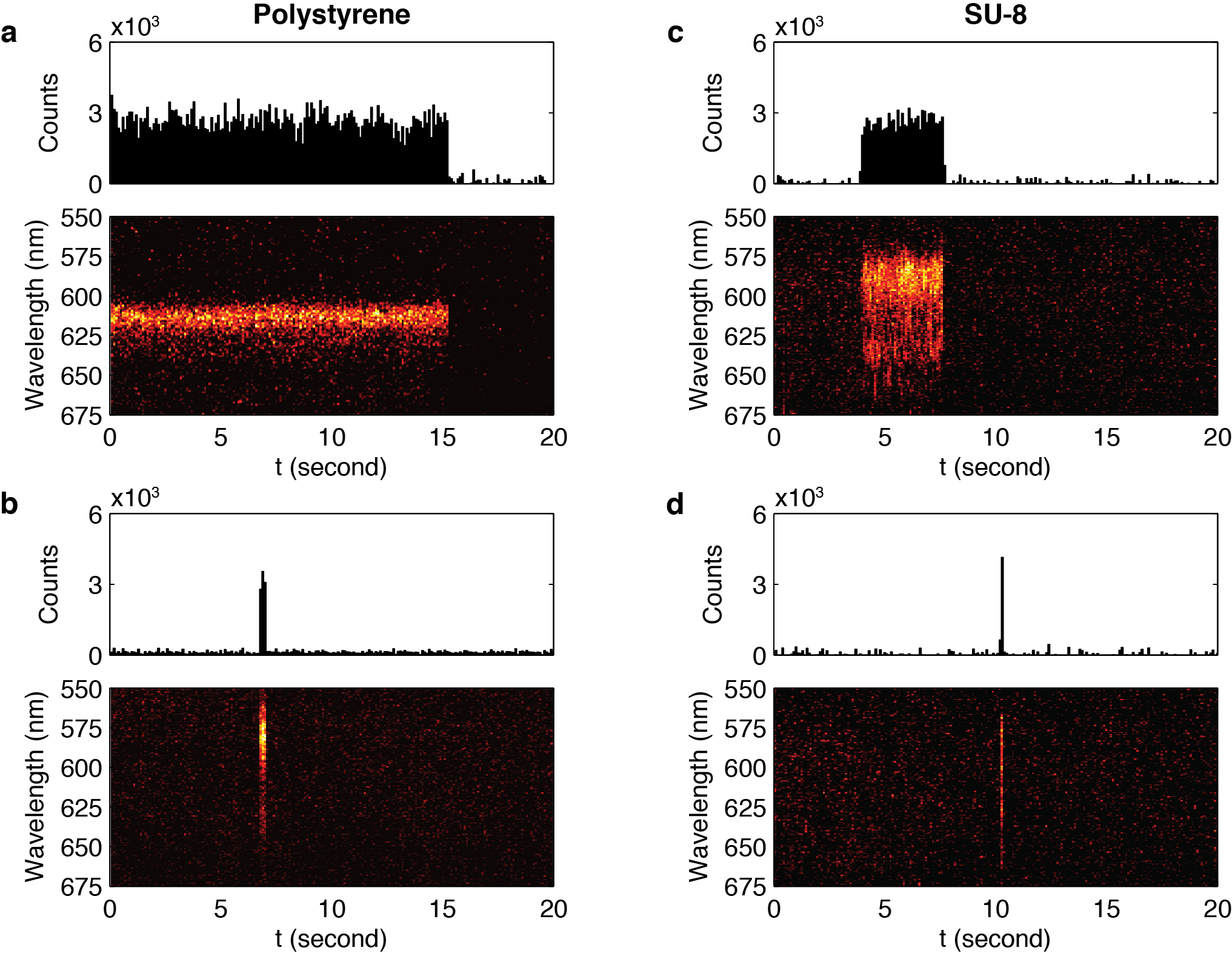


Fig. S3. Long (a) and short (b) lived stochastic blinking events in poly-styrene thin film samples. Counterpart long (c) and short (d) lived blinking events for SU-8 films. Unlike PS and PMMA blinking, SU-8 has similar spectral properties in both long and short lived events.


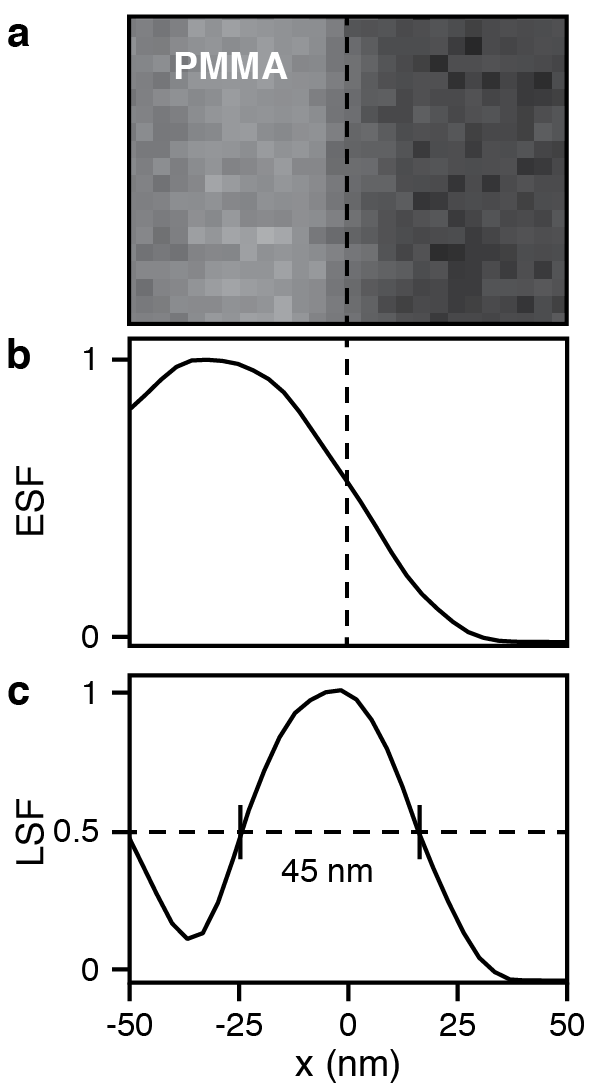


Fig. S4. (A) SEM image shows the edge of a solid PMMA bar used to determine lateral resolution. (B) Edge spread function (ESF) was obtained from its STORM image, whereas (C) shows the line spread function (LSF) calculated from the ESF.


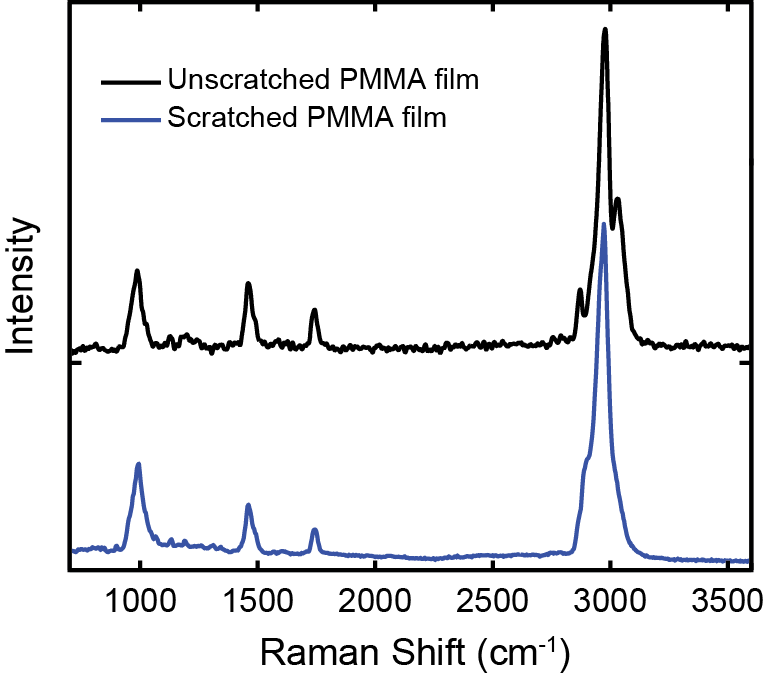


Figure S5. Raman spectra of PMMA thin film. The top-black line displays the Raman spectra from as-deposited undamaged film. The bottom-blue line shows the Raman spectra from a scratched region with increased blinking. Scratched regions display altered peak ratios of the C-H stretch peaks. The changes in spectra Raman spectra after damage suggest that blinking is not only due to sample impurities, but also due to molecular structure changes in the polymer.
